# Amplification of Mdmx and overexpression of MDM2 contribute to mammary carcinogenesis by substituting for p53 mutations

**DOI:** 10.1186/1746-1596-9-71

**Published:** 2014-03-25

**Authors:** Qiong Yu, Yan Li, Kun Mu, Zhishuang Li, Qingyong Meng, Xiaojuan Wu, Yan Wang, Li Li

**Affiliations:** 1 Department of Pathology, Shandong University School of Medicine, 44#,Wenhua Xi Road, 250012 Jinan, Shandong, PR. China; 2 Department of Medical Oncology, Shandong Cancer Hospital and Institute, Jinan University, 250117 Jinan, Shandong, PR. China; 3 The No.2 People’s Hospital of Jinan, 148#, Jingyi Road, 250001 Jinan, Shandong, People’s Republic of China

**Keywords:** Breast cancer, p53, Mdmx, Mdm2, FISH

## Abstract

**Background:**

The p53 tumor suppressor gene is mutated or deleted in nearly half of human cancers. The murine double minute 2 (Mdm2) and Mdmx represent two important cellular regulators of p53. The aim of this study was to evaluate the abnormalities of p53, Mdmx and Mdm2 genes in archived breast cancers.

**Methods:**

We assessed the genetic instability at p53, Mdmx and Mdm2 using high resolution multi-color fluorescent in situ hybridization (FISH) protocol and detected the expression status of the tumor protein p53 (TP53), MDMx and MDM2 by immunohistochemistry in 115 archived samples of infiltrating ductal breast carcinomas with foci of ductal carcinoma in situ (DCIS) components.

**Results:**

The presence of p53 allelic loss and/or TP53 overexpression was observed in 38% out of all patients, and was significantly more often in larger, high grade, ER negative and high ki67 tumors. Mdmx amplification with low-level increase of gene copy number is at high frequency while Mdm2 amplification is rare in primary breast cancer. Mdmx amplification was seen in more invasive carcinomas than preinvasive lesions. MDMx and MDM2 overexpression were detected in 65% and 38% of all cases respectively. Moreover it was showed that most tumors contained either p53 dysfunction or Mdm2 alteration, but not both. This distribution was significant (*P* < 0.05). Inverse correlation between Mdmx amplification/overexpression and p53 disfunction was also observed (*P* < 0.05).

**Conclusions:**

Our results suggest the involvement of Mdm2 and Mdmx in p53-independent breast carcinogenesis and Mdmx may contribute to the regulation of p53 independently of Mdm2.

**Virtual slides:**

The virtual slides for this article can be found here: http://www.diagnosticpathology.diagnomx.eu/vs/1450529994118798.

## Background

The p53 tumor suppressor gene has a central role in maintaining the integrity of the genome and the defense against cancer. The tumor protein 53 (TP53) becomes stabilized and regulates numerous downstream targets to induce cell cycle arrest, senescence, apoptosis, and DNA repair in response to diverse stresses. Mutation and LOH at the p53 locus occur as tumors progress under conditions of increasing genomic instability [1-3]. p53 is mutated in nearly half of all human cancers, and it is functionally abrogated in much of the remaining 50% of cancers through signaling pathways [4]. In breast cancer, approximately about 15% to 50% of the cases carry a mutant p53 gene and/or loss of heterozygosity (LOH) at chromosome location 17p13, where the p53 gene is located [5-7].

TP53 is negatively regulated by numerous factors. The murine double minute 2 (Mdm2) and Mdmx represent two important cellular regulators of p53. The Mdm2 gene was identified as one of three unknown genes (Mdm1-3) coamplified in the spontaneously transformed 3T3-DM mouse cell line [8]. Acting as an ubiquitin (Ub) protein ligase (E3), MDM2 (also called HDM2) can bind and ubiquitinate TP53 and promote rapid degradation of TP53 through the ubiquitin proteolysis pathway, which keeps TP53 at low levels in the absence of stress signals. MDM2 overexpression has been observed in about a third of human sarcomas that retained wild-type TP53 [3,9-11].

MDMx (also known as MDM4), a TP53 binding protein structurally homologous to MDM2, was more recently identified [12]. MDMx was found amplified or overexpressed in 10–20% of breast cancers, glioblastomas, retinoblastomas, and soft tissue sarcomas [3,13-15] in the presence of wild-type TP53, which confirmed that aberrant expression of MDMx may contribute to tumor formation by inhibiting TP53 activity. But the molecular details of the role of MDMx in the control of P53 and tumorigenesis are not well understood.

Although MDM2 and MDMx are overexpressed in many malignancies, data were mainly from cell-based studies and in vivo studies on mouse models. Limited studies using human archived tissue of breast cancers revealed the roles of the above molecular markers in carcinogenesis and the relationship among them as well as the relationship with the clinicopathologic characteristics. And to our knowledge, there was no study on these molecular markers using invasive carcinoma and carcinoma in situ in the same tumors. In present study our aims were to assess the genetic instability at p53 (located on 17p13.1), Mdmx (located on 1q32.1) and Mdm2 (located on 12q15) and address the roles of these proteins in breast cancer progression. Using multi-color fluorescence in situ hybridization (FISH) protocols in 115 primary breast cancers, we examined the genetic changes at p53, Mdmx and Mdm2 loci in archived breast cancers. The expression of these proteins was evaluated by immunohistochemistry.

## Materials and methods

### Patients and tumor characteristics

One hundred and thirty seven primary invasive breast carcinoma samples with foci of ductal carcinoma in situ (DCIS) were collected from 137 women undergoing surgery between January 2007 and September 2008 in Qilu Hospital of Shandong University, Jinan, China. The study was approved by the Ethics Committee of Shandong University. The tumor samples were fixed in 4% phosphate buffered formaldehyde directly after the operation and paraffin embedded. From each specimen ten contiguous sections were prepared and used for hematoxylin and eosin staining, immunohistochemistry and FISH procedure (thickness 4 μm). Reliable immunohistochemistry staining could be obtained from 129 of these tumors and good-quality DNA was available for hybridization of 121 of the 137 tumors. Out of 137 tumors initially selected 22 tumors were excluded for subsequent analysis.

For each tumor, malignancy grade, tumor size (diameter), lymphnode status at the time of diagnosis were evaluated. All tumors were diagnosed according to World Health Organization criteria [16] and graded based on the recommendations of Elson and Ellis.

### Immunohistochemistry

Immunohistochemistry was carried out as described previously [17]. The antibodies against ER (Clone 1D5, DAKO), PR (Clone PgR 636, DAKO), Ki67 (Clone MIB-1, DAKO), HER-2 (4B5, Ventana), p53 (DO-1, Santa Cruz), MDM2 (SMP14, Santa Cruz), MDMx (ab76362, abcam) were applied at dilution 1:100, 1:100, 1:1000, 1:100, 1:200 and 1:800. A positive section produced in preliminary experiments was used as a positive control. Normal fetus serum was used as a negative control by replacement of the relevant primary antibody.

Only distinct nuclear staining was accepted as positive reactions for ER, PR, p53 and ki67, whereas HER-2 showed a membrane staining. It was scored positive for ER or PR if > =1% of the nuclei of neoplastic cells showed definitive staining. HER-2 was evaluated according to ASCO/CAP guideline proposed for the evaluation of HER2 testing: a 3+ score or a 2+ score with FISH ratio of more than 2.2 was considered to indicate positive HER2 expression. Only tumor tissues with diffuse strong nuclear staining for TP53 was considered to show mutant p53 while scattered weak to moderate nuclear staining was scored negative [18,19]. Ki67 status was scored low if <15% of the nuclei of neoplastic cells were positive, and high if > =15% of the nuclei of neoplastic cells were positive [20]. All tumor cells with nuclear staining or simultaneous nuclear and cytoplasmic staining were regarded as positive for MDM2 and MDMx. The degree of staining of tumor cells was categorized as –, negative; +, weak; ++, moderate; and +++, strong. Normal breast tissues were included as the controls.

### FISH procedure

High resolution multi-color FISH was performed to detect gene copy number changes of p53, Mdmx and Mdm2 in all 115 cases. All probes were obtained from Professor Anders Zetterberg, Cancer Center Karolinska, Sweden, and labeled with Spectrum Green, Spectrum Orange or Texas Red. Sections were removed excess wax followed by dehydration in absolute alcohol. Antigenic recovery was performed through incubating the slides for 1 hour at 80°C in 0.1 M citrate buffer (pH 6.0). A 10-minute digestion with pepsin (1 mg/ml in 0.01 M HCL) was performed followed by fixation in 1% PF. The probes were dissolved in the hybridization mixture. Denaturation of probes and target DNA were performed simultaneously at 90°C for 10 min and each slide was incubated in a moist chamber for hybridization at 47°C overnight. After hybridization, slides were washed in 4 × SSPE for 10 min at 37°C and 47°C respectively. Nuclei were mounted and counterstained with 4′,6-diamino-2-phenylindole (DAPI, Vectashield).

### Signal scoring and evaluation

Signal evaluation was carried out using an Axioplan 2 microscope (Carl Zeiss AB, Sweden) mounted with a charge-coupled device (CCD) camera Axiocam MRM (Carl Zeiss AB), coupled with a computer with Axio Vision software (Carl Zeiss AB). Only signals in the tumor areas based on both a consecutive section stained by hematoxylin and eosin and DAPI morphology were counted and evaluated. Two researchers (Yu&Li) carried out all investigations independently. In cases of discordance, the results would be evaluated by the third observer (Li Li) and the result which two observers supported would be taken.

The exact copy number of the signals per nucleus was recorded, and at least 100 non-overlapping nuclei per sample were analyzed. More than 30% of counted nuclei with the number of signals less than those of corresponding chromosomes defined those samples containing gene deletion. Gene amplification was defined by the presence of an excess in the number of gene loci over the number of corresponding chromosomes on more than 20% of counted cells [21]. The chromosome 1,12,17 centromericprobe was used as internal controls.

### Statistic analysis

Statistical analyses were performed using the SPSS for Windows version 16.0. The correlations between variables were performed using Fisher’s exact test or Spearsman rank correlation test. All tests were two-sided with significance level α set to 0.05.

## Results

### Characteristics of patients

The patients were all female and their ages at the time of the diagnosis ranged from 23 to 85 years (mean 49 years). The tumor size ranged from 0.6 to 11 cm in the greatest dimension (mean 2.8 cm). Patients and tumor characteristics of 115 cases are summarized in Table 1.

**Table 1:** Alterations of Mdmx, Mdm2 and p53 in 115 primary breast cancers

**Characteristics**	**Patients no. (%)**	**Mdmx amplification**		**Mdm2 overexpression**		**p53 disfunction**	
		**no. (%)**	***P***	**no. (%)**	***P***	**no. (%)**	***P***
Tumor size^1^	36 (31.3%)	22 (61.1%)		19 (41.3%)		8 (22.2%)	
d≦20 mm	66 (57.4%)	35 (53.0%)	0.710	21 (31.8%)	0.301	29 (47.5%)	0.009
50 mm > =d > 20 mm	13 (11.3%)	8 (61.5%)		4 (30.8%)		7 (53.8%)	
d > 50 mm							
Grade							
I	9 (7.8%)	7 (77.8%)		5 (55.6%)	0.738	0 (0)	0.001
II	67 (58.3%)	38 (56.7%)	0.244	22 (32.8%)		22 (32.8)	
III	39 (33.9%)	20 (51.3%)		17 (43.6%)		22 (56.4%)	
Nodal status^2^					0.122		0.348
N	51 (44.3%)	31 (60.8%)	0.453	24 (47.1%)		18 (35.3%)	
N+	64 (55.7%)	34 (53.1%)		20 (31.2%)		26 (40.6%)	
ER status					0.116		0.016
Negative	42 (36.5%)	24 (57.1%)	1.000	12 (28.6%)		22 (52.4%)	
Positive	73 (63.5%)	41 (56.2%)		32 (43.8%)		22 (30.1%)	
PR status	4				0.241		0.185
Negative	5 (39.1%)	22 (48.9%)	0.248	14 (31.1%)		20 (44.4%)	
Positive	70 (60.9%)	43 (61.4%)		30 (42.9%)		24 (34.3%)	
HER-2 status					0.330		0.305
Negative	93 (80.9%)	54 (58.1%)	0.633	38 (40.9%)		37 (40.7%)	
Positive	22 (19.1%)	11 (50.0%)		6 (27.3%)		7 (31.8%)	
Ki67 expression					1.000		0.006
Low	44 (38.3%)	28 (63.6%)	0.251	17 (38.6%)		10 (22.7%)	
High	71 (61.7%)	37 (52.1%)		27 (38.0%)		34 (47.9%)	
Mdmx amplification					0.176		0.033
Yes	65 (56.5%)	___	___	21		19	
No	50 (43.5%)			23		25	
Mdm2 overexpression		___	___	___	___		0.018
Yes	44 (38.3%)					11	
No	71 (98.3%)					33	

^1^d, diameter.

^2^N0, node metastasis negative; N+, node metastasis positive.

### Genetic instability in primary breast cancer

**Figure 1:**
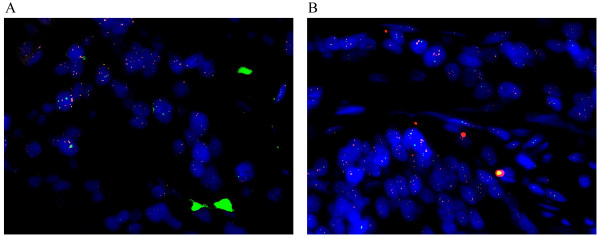
**Genetic abnormalities detected by multi-color FISH in primary breast cancer.** p53, Mdmx and Mdm2 were labeled with green, orange and red respectively **(A)** Low level of Mdmx amplification accompanied with normal gene copy number of p53 and Mdm2 is shown, most cancer cell nuclei have <10 orange signals (FISH,×630). **(B)** The tumor cells lack of gene number changes of Mdmx and Mdm2 show LOH of p53, most cancer cell nuclei have only 1 green signal (FISH,×630).

Mdmx and Mdm2 amplification and p53 deletion were identified by multi-color FISH. As shown in Table 1, among the 115 breast cancers amplification of Mdmx was found in 65 cases (56.5%) of cases, higher than published studies [15]. But only low-level increase of gene copy number (<10 copies) was observed in all samples (Figure 1A). Amplification of Mdm2 was identified only in two of these samples (1.7%) indicating that Mdm2 amplification is uncommon in primary breast cancers. Allelic loss of p53 was detected in 28/115 (24.3%) of cases (Figure 1B). The frequency of Mdmx and/or p53 genetic abnormality was up to 70% (81/115), which indicated most tumors showed either p53 or Mdmx genetic abnormalities. No significant correlation was observed between p53 deletions and Mdmx amplification (Table 2).

**Table 2:** Association analyses of Mdmx, Mdm2 and p53 genetic changes in primary breast cancers

	**Mdmx amplification**			**Mdm2 amplification**		
	**Yes**	**No**	***P***	**Yes**	**No**	***P***
p53 deletion						1.000
Yes	12	16	<0.125	0	28	
No	53	34		2	85	

**Figure 2:**
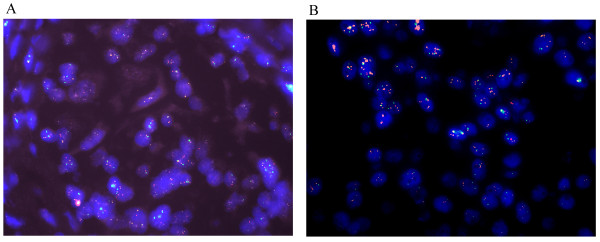
Mdmx amplification in the infiltrating components (A) but not in the DCIS (B) (FISH,×630).

We compared gene copy number changes between DCIS and invasive areas, and found 56 cases showed Mdmx amplification in both DCIS and invasive areas and 9 cases were observed Mdmx amplification only in infiltrating areas but not in the DCIS components (Figure 2). p53 deletion was found in from in situ to invasive disease for all of the gene loss cases thus supporting earlier findings that p53 abnormality has a role early in the pathogenesis of breast lesions.

### The expression status of TP53, MDMx and MDM2 proteins

**Figure 3:**
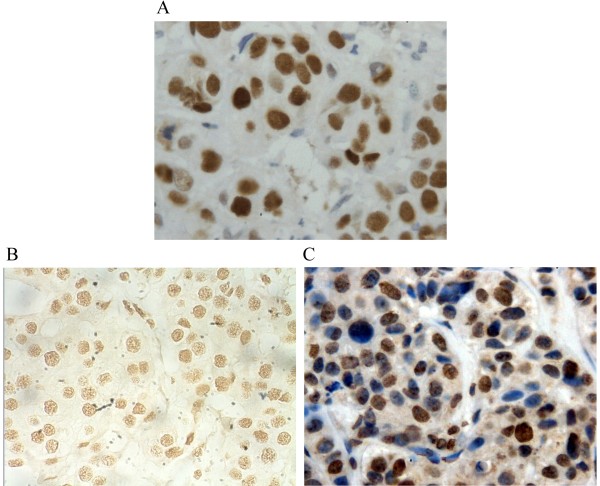
**Immunohistochemical staining for TP53,MDMx and MDM2 in primary breast cancer. (A)** p53 accumulation with diffuse strong nuclear staining in the tumor cells (×400). **(B)** MDMx overexpression with diffuse and strong nuclear staining (×400) in the tumor cells with Mdmx amplification. **(C)** MDM2 overexpression with nuclear and cytoplasmic staining in the tumor cells (×400).

We analyzed TP53, MDMx and MDM2 proteins levels in formalin-fixed tissues of all 115 breast cancer samples using an immunohistochemical approach. In total, 20 tumors (17.3%) showed diffuse strong staining for TP53 (Figure 3A). 75 tumor (65.2%) showed MDMx diffuse strong staining (scored +++) (Figure 3B), which correlated with Mdmx amplification (p < 0.01). Herein, MDMx overexpression was recognized as diffuse strong staining (scored +++). MDM2 overexpression was detected in 44 out of 115 cases (38.3%) (Figure 3B). Both cytoplasmic and nuclear compartments were stained with MDMx and MDM2.

### Dysfunction of p53 in relation to Mdmx amplification and MDM2 overexpression

The p53 dysfunctional genophenotype was identified in individual tumors by the presence of LOH of p53 by FISH, p53 protein overexpression by immunohistochemistry, or both. Totally, the presence of LOH of p53 and/or TP53 overexpression was observed in 44 tumors (38.3%) out of all patients. LOH of p53 and its overexpression were each present in around 20% of the cases implying p53 mutations and allelic loss contributed p53 dysfunction equally in this group. They were observed in the same tumor in 4 cases supporting the findings by others that mutation of one p53 allele could be accompanied by loss of the complementary wild type allele [22]. There was inverse correlation between p53 abnormality and MDM2 overexpression (*P* < 0.05). We also found inverse correlation between p53 abnormality and amplification/overexpression of Mdmx (*P* < 0.05).

### Abnormalities of p53, Mdmx and Mdm2 in relation to tumor clinicopathologic characteristics

The dysfunctional p53 phenotype was found to be linked with tumor size (*P* < 0.05) and tumor grade (*P* < 0.01). In addition, tumors with dysfunctional p53 preferentially had negative ER status (*P* < 0.05) and high ki67 index (*P* < 0.01). But Mdmx amplification or MDM2 overexpression was not found to be correlated with any of the above clinicopathologic tumor characteristics. A detailed summary of the data is summarized in Table 1.

## Discussion

Breast cancer is characterized by a number of genetic aberrations [23-25]. Although improvements have been achieved in recent years, few genetic biomarkers are available to easily identify individuals at risk for breast cancer or breast cancer progression [26-28]. A better understanding of the molecular mechanisms involved in breast cancer initiation and progression will likely contribute to providing useful prognostic biomarker and therapeutic target for breast cancer therapy.

FISH is considered to be the most precise method for amplification and deletion detection. In this study, we successfully detected the gene copy number changes of p53, Mdmx and Mdm2 by multi-color FISH, which enables investigators obtaining far more information from one specific cell at one time, rather than carrying out separate experiments on multiple specimens prepared from the same sample, then extrapolating results.

Dysfunction of p53 often occurs as a result of mis-sense mutation, but can also result from nonsense or deletion mutation [3]. TP53 is a product of the mutated gene. Therefore here TP53 overexpression using immunohistochemical method was adopted to investigate p53 mutation. For scattered weak to moderate staining can be observed in normal breast tissue, hyperplasia breast tissue and tumor tissue without p53 mutation, only tumor tissues with diffuse strong nuclear staining for TP53 was considered to show mutant p53 as in serious papillary carcinoma in ovary. In this study, FISH was adopted to detect p53 allelic loss as a co-criterion for identifying the dysfunctional p53 phenotype. Allelic loss of p53 was detected in 23% of all analyzed samples in present study while 17% of cases showed TP53 overexpression indicating p53 mutation. The presence of p53 allelic loss and/or TP53 overexpression was observed in 38% out of all patients, within the range of frequencies reported by others [29,30]. Because of the involvement of DCIS, p53 alteration must be an early event in breast carcinogenesis. According to previous reports, mutation of p53 is associated with increased tumor size and tumor grade, axillary lymph node metastases and ki67 expression [30-32]. But data presented here indicate there was no significant relationship between p53 allelic loss or TP53 overexpression and tumor node status, whereas p53 dysfunction was detected significantly more often in larger (d > 20 mm), high grade (grade 3), ER negative and high ki67 index tumors.

LOH and mutation of p53 only present in part of breast cancers. It is believed that tumors retaining wild type p53 contain abnormalities in other genes that interact with p53 or are downstream of p53 and result in an identical physiological defect within the cells. One of the best examples of the latter class is amplification or overexpression of Mdm2, which was observed in a subset of human tumors, some of which retain wild-type p53 [33] leading to the conclusion that increased levels of MDM2 directly contribute to human tumor formation by substituting for mutations in p53 gene, which represents an alternative mechanism by which tumor cells escape from the tumor suppressive activities of p53. Overexpression of MDM2 can impair wild-type TP53 function in two ways by binding to the transactivation site of TP53 in the nucleus or by targeting TP53 for ubiquitination and degradation in the cytoplasm, and linked with low levels of TP53 immunostaining in human breast cancers [34]. Frequent overexpression of MDM2 in advanced breast tumors was observed [34]. It was reported MDM2 protein overexpression often due to gene amplifications [35,36]. But Mdm2 amplification was found only in two samples while its overexpression was observed in 38% of all tumors in this study, which supported Mdm2 amplification occurs at a lower frequency than increased transcription or enhanced translation in breast cancers. So in this study MDM2 overexpression was used to reflect Mdm2 abnormality. Importantly, we found most tumors contained either a TP53 alteration or a MDM2 alteration, but not both. This distribution was significant (*P*< 0.05), and strongly suggests alterations of these two genes are mutually exclusive. Our data support the hypothesis that the major effect of MDM2 overexpression is identical to that resulting from p53 mutation. One would expect that either TP53 or MDM2 would be altered and alterations of both genes should be examined in a given breast cancer. There were few tumors observed without TP53 dysfunction and MDM2 overexpression in this study. So there might be other alterations of TP53 or MDM2, or some of these tumors might progress through genetic events that involve a totally different pathway. More detailed analyses of such tumors are needed to reveal.

Several studies together now also implicate aberrant expression of MDMx could thus contribute to tumor formation [37]. Unlike MDM2, MDMx does not have intrinsic E3 ligase activity and does not promote TP53 degradation. However, MDMx binds TP53 in its transactivation domain and is thereby able to inhibit its transcriptional activity. Amplification of Mdmx was found in several tumor types. Migliorini found amplification of Mdmx correlated with a wild-type p53 status and lack of Mdm2 amplification [38]. In this study Mdmx was amplified in 57% of all cases, while it was overexpressed in 65% of all tumors, indicating MDMx overexpression was mainly due to its amplification. Mdmx amplification was adopted in data analyses. Although not all the tumors with Mdmx amplification show wild type p53, significant inverse correlation between Mdmx amplification and TP53 overexpression was still observed. We did not find correlation between Mdmx amplification and MDM2 overexpression, but tumors with Mdmx amplification were more likely lack of MDM2 overexpression. Up to now, it was still controversial whether each plays its own distinct role or MDM2 and MDMx function together as one heterocomplex in p53 regulation. Our data seems to support MDMx may contribute to the regulation of TP53 independently of MDM2. A recent evidence demonstrated that endogenous level of MDMx could regulate transformation and chromosomal stability in TP53-deficient cells and these MDMx functions were not shared by MDM2, and were distinct from the well-established ability of MDMx to complex with and inhibit TP53 activity [39]. Together these data strongly indicate that Mdmx amplification is a common event in breast carcinogenesis, even in most tumorigenesis and MDMx probably functions as an oncogene through a quite different way compared to MDM2. The molecular mechanism that prevents TP53 activation and carcinogenesis in the presence of high level of MDMx is largely unknown, which need to be elucidated in the future. Furthermore we found Mdmx amplification was seen in more invasive breast cancers. So we speculate amplified Mdmx more likely to be associated with tumor progression. Larger sample sizes to provide more definitive data on the potential role of genetic changes of Mdmx in breast cancer progression should be performed in the future.

## Conclusions

In conclusion, we detected genetic alterations of p53, Mdmx and Mdm2 by multi-color FISH in one specific cell simultaneously and compared these genetic alterations in DCIS and invasive lesions in individual cases for the first time. Our data indicate that disruption of the TP53 pathway by high levels of MDM2 and MDMx are common events in the breast tumorigenesis and Mdmx amplifies quite frequently with low-level increase of gene copy number while few tumors with Mdm2 amplification are seen in primary breast cancers. Mdmx amplification is seen in invasive breast cancer but not in DCIS in some cases and likely to be associated with tumor progression. Moreover, high levels of both genes in breast cancer that retain wild-type p53 suggest that these inhibitors may substitute for mutations in p53, therefore, contribute to the severity and progression of the disease. But as an oncogene, high level of MDMx may act through a quite different way compared to MDM2. Together, our data strongly suggest that overexpression of MDM2 or MDMx and p53 mutations in primary breast cancer are mutually exclusive events. The presence of high levels of MDM2 and MDMx in many breast cancers suggests that these data should be considered in the treatment of breast cancer.

## Competing interests

We declare that we have no conflict of interests.

## Authors’ contributions

LL designed the study, carried out the experiments and drafted the manuscript; QY, YL, ZSL, KM carried out the FISH detection. XJW and YW performed immunohistochemical interpretation of the tumor tissue. QYM participated in the statistical analysis. All authors read and approved the final manuscript.
